# Systemic treatment and radiotherapy, breast cancer subtypes, and survival after long-term clinical follow-up

**DOI:** 10.1007/s10549-019-05142-x

**Published:** 2019-02-12

**Authors:** Sherry X. Yang, Eric C. Polley

**Affiliations:** 1 Division of Cancer Treatment and Diagnosis, National Cancer Institute, National Institutes of Health, Bethesda, MD USA; 2 Division of Biomedical Statistics and Informatics, Department of Health Sciences Research, Mayo Clinic, Rochester, MN USA

**Keywords:** Breast cancer subtypes, Hormone receptor (HR), HER2, Overall survival, Radiotherapy, Systemic treatment, Triple-negative breast cancer

## Abstract

**Background:**

It remains unclear whether breast cancer subtypes are associated with clinical outcome in patients without any treatment including systemic and radiation therapy as an independent entity. Understanding the survival profiles among subtypes by treatment status could impact optimal selection of treatments.

**Methods:**

Patients were diagnosed with invasive breast cancer from the community hospitals across four geographical regions of the United States. Expression of hormone receptor (HR) and HER2 in tumor specimens from 1169 patients was centrally determined by immunohistochemistry and fluorescence in situ; breast cancer was classified into HR^+^/HER2^−^, HR^+^/HER2^+^, triple-negative, and HER2^+^ subtypes. Overall survival (OS) at a median follow-up of about 15 years among subtypes in untreated patients and those with systemic treatments and radiotherapy was analyzed by Kaplan–Meier method and multivariable analysis adjusting for age, tumor size and grade, number of positive nodes, stage and breast cancer subtypes.

**Results:**

Without treatment, breast cancer subtypes were not associated with OS (*P* = 0.983) and remained insignificant for prognosis by multivariable analysis after adjusting for confounders. This contrasted with a significant survival difference across the subtypes in patients with conventional therapies (*P* < 0.0001). Compared with HR^+^/HER2^−^ subtype, triple-negative subtype (HR 1.5, 95% CI 1.11–2.04; *P* = 0.009) and HER2^+^ subtype (HR 2.18, 95% CI 1.48–3.28; *P* = 0.0001) were significantly associated with worse survival by multivariable analyses.

**Conclusion:**

Breast cancer subtypes are not associated with survival in untreated patient population and, in contrast, significantly associated with prognosis in patients with conventional therapy. The data provide evidence of treatment-associated differential outcomes among breast cancer subtypes.

**Electronic supplementary material:**

The online version of this article (10.1007/s10549-019-05142-x) contains supplementary material, which is available to authorized users.

## Background

Breast cancer accounts for about a quarter of all cancers and 15% of cancer-specific deaths in women globally [1]. It is the most frequently diagnosed and second leading cause of cancer mortality for women in the United States, with about 260,000 new incidences and approximate 40,000 deaths recorded each year [2]. About 13–41% of patients with operable stage I, II and III breast cancer experience distant and local relapses, and eventually succumb to their disease [3, 4]. Hormone receptor (HR) and human epidermal growth factor receptor 2 (HER2) are integrated into clinical management and lately prognostic staging of breast cancer [5–7]. HR (estrogen receptor α, ER^+^ and/or progesterone receptor, PR^+^)-positive breast cancer is consisted of 65–80% of all breast cancers, which are routinely managed by 5 years of endocrine therapy that is extended up to 10 years in recent years [8–10]. Patients with high risk factors such as HER2-positivity (HER2^+^), HR-negative (HR^−^) status or positive lymph nodes are recommended for cytotoxic chemotherapy [8, 11]. Radiation therapy is recommended for all patients who undergo breast cancer conserving surgery and may be used for those either with a cancer larger than 5 cm or node-positive disease after mastectomy.

The intrinsic gene expression signatures in breast cancer were discovered by DNA microarray technology that classifies breast cancer into the molecular subtypes predominantly as luminal A, luminal B, HER2-enriched and basal-like [12]. Largely in agreement with the gene expression profiling data, breast cancer is categorized into HR^+^/HER2^−^ (ER^+^, PR^+^ and HER2^−^ or luminal-A), HR^+^/HER2^+^ (ER^+^, PR^+^ and HER2^+^ or luminal-B), HER2^+^ (HER2^+^, ER^−^ and PR^−^ or HER2-enriched), and triple-negative breast cancer (TNBC or ER^−^, PR^−^ and HER2^−^ or basal-like) subtypes by established immunohistochemistry (IHC) classifier [13]. The IHC-based classification with joint HR and HER2 status is the mostly implemented method for establishing breast cancer subtypes in the clinic [14]. The molecular subtypes were found to provide prognostic information, with luminal subtypes exhibiting better clinical outcomes [15, 16]. The St. Gallen expert consensus panel has adopted a subtype-based approach in the context of current treatment modalities of early stage breast cancer [17]. However, some studies revealed that intrinsic subtyping was not prognostic nor predictive of response to treatments [18]. In a large cohort study, there was no significant difference in survival according to molecular subtype 5 years after diagnosis until ~ 20 years of clinical follow-up [19]. The gene expression signature established from most of the patients receiving radiotherapy and conventional therapy cannot predict outcomes of untreated patients [20, 21].

Currently, it remains unclear whether the molecular subtype has prognostic significance in patients who did not undergo any type of therapy including systemic treatments and radiation therapy except surgery. Thus, there is a heightened need to evaluate outcome by subtype in women with and without multimodality therapy within a population separately. In this investigation, our study objectives are the overall survivals (OS) by treatment status among breast cancer subtypes using Kaplan–Meier and multivariable Cox regression analyses adjusting for confounding factors.

## Methods

### Patient population and breast cancer subtypes

The study patient population was consisted of women diagnosed with invasive breast cancer with stage I, II or III from 1985 to 1997 in the community hospitals of four geographical regions of the United States as described previously [22, 23]. They participated in the accreditation program of the Commission on Cancer of the American College of Surgeons. The project received full review and approval by institutional review board at each participating site [22]. The coded dataset established was centrally maintained, which includes age, clinical and pathological variables, types of treatment received, vital status, and clinical follow-up for a maximum of 282 months (23.5 years) [22]. All 1169 patients represented by the Cooperative Breast Cancer Tissue Resource (CBCTR), but one, underwent surgery for primary treatment of their disease. This resulted in 1168 participants including 372 patients who did not receive treatments, and 796 with endocrine therapy, chemotherapy and radiation therapy alone, and/or in combination. No patients received trastuzumab treatment in the population.

Breast cancer subtypes were determined by HR status including ER and PR, and HER2 status, which were centrally assayed and scored by the CBCTR pathologists according to the American Society of Clinical Oncology (ASCO) and the College of American Pathologists (CAP) guidelines [24, 25]. ER/PR was assessed as positive if ≥ 1 tumor cells stained. HER2 positivity was defined as IHC Score 3+, IHC Score 1+ or 2+ or IHC not available and FISH amplified. The four major breast cancer subtypes defined by immunohistochemistry and fluorescence in situ hybridization (FISH) were HR^+^/HER2^−^ and HR^+^/HER2^+^ subtypes; HER2^+^ subtype was defined as having HER2^+^ and HR^−^ status; and triple-negative breast cancer was referred to the tumors with all three markers being negative (TNBC; HER2^−^/ER^−^/PR^−^). Of 1168 patients, ~ 16% were unclassified due to a lack of ER/PR and HER2 data and were excluded for clinical outcome analysis. This study on the de-identified human tumor specimen/dataset was received approval from the Office of Human Research Protections, National Institutes of Health (Bethesda, Maryland). The study complies with the REMARK reporting recommendations for tumor marker studies [26].

### Statistical analysis

Chi-squared test of association was used to compare categorical variables among breast cancer subtypes by treatment status. Length of follow-up for OS was defined as number of months from the date of diagnosis to the date of death due to any cause, or to the date last known alive. Assessment of time to survival event interval used the Kaplan–Meier method and the log-rank test for association. Cox’s proportional hazards method was used for multivariable models including age at diagnosis, year of diagnosis, histology type, tumor size and grade, and number of positive nodes, TNM stage, and/or breast cancer subtypes. A *P* value less than 0.05 was considered statistically significant. All statistical analysie were performed in R (R Foundation).

## Results

### Patient and clinicopathologic factors, and breast cancer subtypes by treatment status

Of 1169 participants, 986 (84%) were successfully classified into the breast cancer subtypes by the established IHC/FISH classifier. The median age of 986 participants was 60 years (range 25 to 96 years). There were 301 patients who did not receive any treatment, and 685 that received either endocrine therapy (164 patients), chemotherapy (138 patients), and radiation therapy (94 patients) alone or in combination (289 patients). Table 1 summarized the distribution of demographic and clinicopathologic characteristics by subtype in the untreated patients. More women with HR^+^/HER2^−^ (83%) and HER2^+^ (84.6%) tumors were older than 50 years relative to those with triple-negative (67.4%) and HR^+^/HER2^+^ (66.7%) subtypes (*P* = 0.047). TNBC subtype had 67.4% and HER2^+^ category had 69.2% of grade III tumors, in contrast to 12.9% for HR^+^/HER2^−^ and 28.6% for HR^+^/HER2^+^ subtypes (*P* < 0.001). At diagnosis, about 46% of HER2^+^ subtype had positive lymph node status, compared to ~ 15% for HR^+^/HER2^−^, ~ 24% for HR^+^/HER2^+^, and ~ 23% for TNBC subtypes (*P* = 0.025).

**Table 1:** Age and clinicopathologic variables at diagnosis by breast cancer subtype in untreated patients

Variable/subtype	Total (*n* = 301)	HR^+^/HER2^−^ (*n* = 224)	HR^+^/HER2^+^ (*n* = 21)	TNBC (*n* = 43)	HER2^+^ (*n* = 13)	*P* value*
	No. (%)	No. (%)	No. (%)	No. (%)	No. (%)	
Age at diagnosis						0.047
≤ 50	61 (20.3%)	38 (17.0%)	7 (33.3%)	14 (32.6%)	2 (15.4%)	
> 50	240 (79.7%)	186 (83.0%)	14 (66.7%)	29 (67.4%)	11 (84.6%)	
Histology						0.344
Ductal	267 (88.7%)	195 (87.1%)	20 (95.2%)	39 (90.7%)	13 (100%)	
Lobular	34 (11.3%)	29 (12.9%)	1 (4.76%)	4 (9.3%)	0 (0%)	
T stage						0.793
T1	226 (75.1%)	172 (76.8%)	17 (81%)	29 (67.4%)	8 (61.5%)	
T2	52 (17.3%)	37 (16.5%)	3 (14.3%)	9 (20.9%)	3 (23.1%)	
T3	16 (5.32%)	10 (4.46%)	1 (4.76%)	4 (9.3%)	1 (7.69%)	
T4	7 (2.33%)	5 (2.23%)	0 (0%)	1 (2.33%)	1 (7.69%)	
N stage						0.076
N0	241 (80.1%)	186 (83%)	15 (71.4%)	33 (76.7%)	7 (53.8%)	
N1	55 (18.3%)	34 (15.2%)	5 (23.8%)	10 (23.3%)	6 (46.2%)	
N2	5 (1.66%)	4 (1.79%)	1 (4.76%)	0 (0%)	0 (0%)	
Tumor size						0.378
≤ 2	226 (75.1%)	172 (76.8%)	17 (81.0%)	29 (67.4%)	8 (61.5%)	
2–5	57 (18.9%)	42 (18.8%)	3 (14.3%)	9 (20.9%)	3 (23.1%)	
> 5	18 (5.98%)	10 (4.46%)	1 (4.76%)	5 (11.6%)	2 (15.4%)	
N of positive nodes						0.025
0	241 (80.1%)	186 (83.0%)	15 (71.4%)	33 (76.7%)	7 (53.8%)	
1–3	39 (13.0%)	25 (11.2%)	3 (14.3%)	7 (16.3%)	4 (30.8%)	
4–9	10 (3.3%)	4 (1.8%)	1 (4.8%)	3 (7.0%)	2 (15.4%)	
≥ 10	11 (3.7%)	9 (4.0%)	2 (9.5%)	0 (0%)	0 (0%)	
Tumor grade						< 0.001
I	92 (30.6%)	86 (38.4%)	5 (23.8%)	1 (2.33%)	0 (0%)	
II	136 (45.2%)	109 (48.7%)	10 (47.6%)	13 (30.2%)	4 (30.8%)	
III	73 (24.3%)	29 (12.9%)	6 (28.6%)	29 (67.4%)	9 (69.2%)	
Stage						0.361
I	197 (65.4%)	153 (68.3%)	14 (66.7%)	25 (58.1%)	5 (38.5%)	
II	80 (26.6%)	56 (25%)	5 (23.8%)	13 (30.2%)	6 (46.2%)	
III	24 (7.97%)	15 (6.7%)	2 (9.52%)	5 (11.6%)	2 (15.4%)	
ER status						< 0.001
Negative	71 (23.6%)	15 (6.7%)	0 (0%)	43 (100%)	13 (100%)	
Positive	230 (76.4%)	209 (93.3%)	21 (100%)	0 (0%)	0 (0%)	
PR status						< 0.001
Negative	90 (29.9%)	28 (12.5%)	6 (28.6%)	43 (100%)	13 (100%)	
Positive	211 (70.1%)	196 (87.5%)	15 (71.4%)	0 (0%)	0 (0%)	
HER2 status						< 0.001
Negative	267 (88.7%)	224 (100%)	0 (0%)	43 (100%)	0 (0%)	
Positive	34 (11.3%)	0 (0%)	21 (100%)	0 (0%)	13 (100%)	

*CI* confidence interval, *ER* estrogen receptor, *HER2* human epidermal growth factor receptor 2, *HR* hazard ratio, *PR* progesterone receptor, *No*. number, *OS* overall survival, *TNBC* triple-negative breast cancer

*P < 0.05 was considered statistically significant

Within the patient population receiving conventional treatments, the distribution of age and clinicopathologic variables by subtype were shown in the Supplementary Table 1. There was a significant variation of tumor sizes or T stage among subtypes. The age, tumor grade, and number of positive nodes remain significantly different across the subtypes as the untreated patient group.

### Survival by subtype with and without treatments

**Fig. 1:**
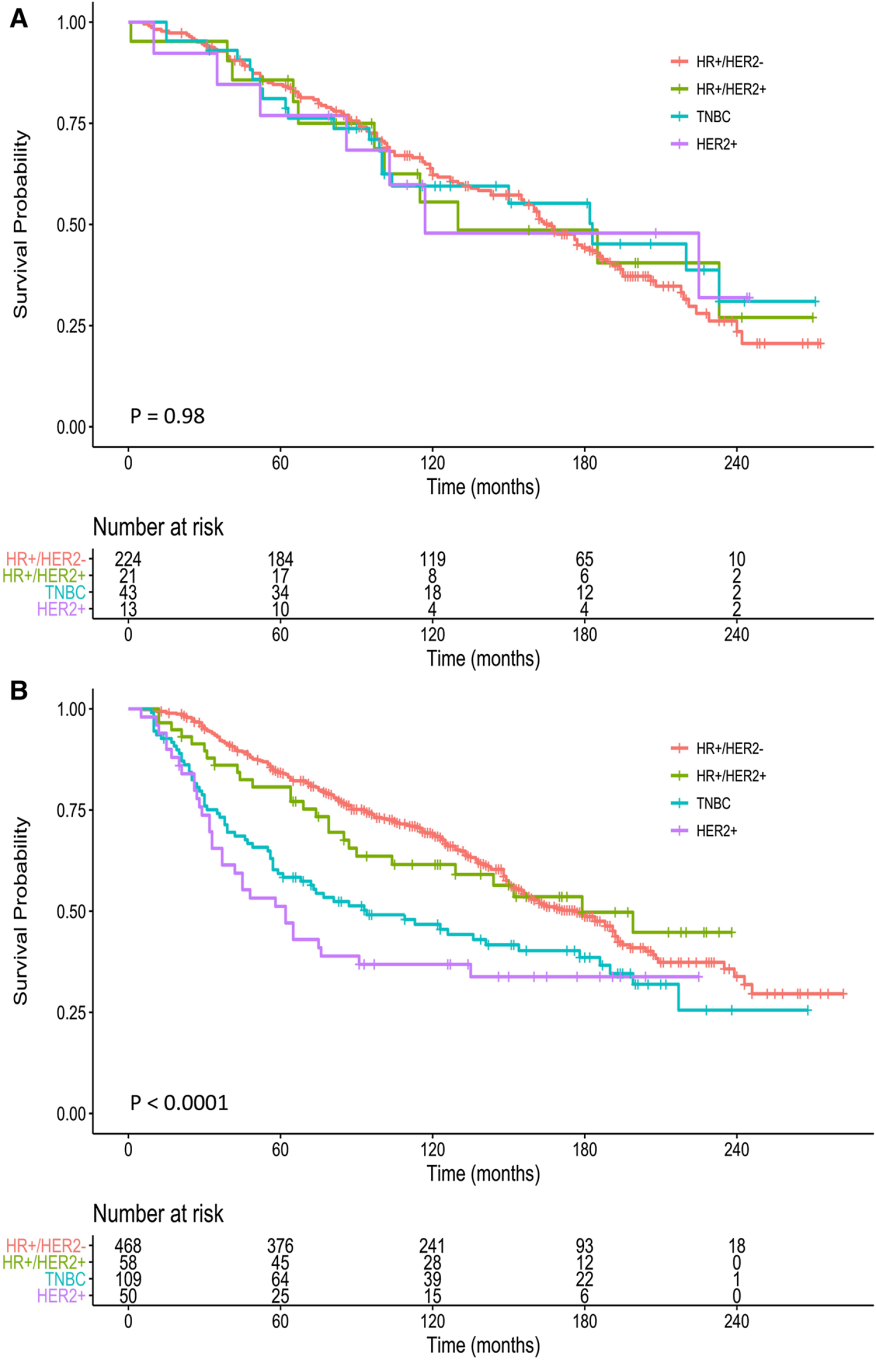
Overall survival among breast cancer subtypes by treatment status. Kaplan–Meier analysis of the survival in the absence of treatment among HR^+^/HER2^−^, HR^+^/HER2^+^, TNBC, and HER2^+^ subtypes (**a**); and the survival rates in patients with HR^+^/HER2^−^, HR^+^/HER2^+^, TNBC, and HER2^+^ subtypes undergoing conventional therapy (**b**). HR^+^/HER2^−^, hormone receptor-positive and HER2-negative; HR^+^/HER2^+^, hormone receptor-positive and HER2-positive; TNBC, triple-negative breast cancer

The median follow-up for OS was 177 months or 14.75 years (range of 1 to 282 months or 0.08 to 23.5 years) in the patient population. We evaluated the association between breast cancer subtypes and prognosis separately by treatment status through both univariate and multivariable analyses (Fig. 1; Tables 2, 3).

**Table 2:** Multivariable Cox’s regression analyses for OS in 301 untreated patients

Variable	Adjusted HR	95% CI	*P* value*
HR^+^/HER2^+^	1.1	(0.55–2.07)	0.84
TNBC	0.68	(0.40–1.18)	0.17
HER2^+^	0.69	(0.29–1.58)	0.37
Age at diagnosis > 50	2.22	(1.35–3.64)	0.0017
Histology lobular	1.36	(0.84–2.20)	0.21
T Stage			
T2	1.71	(0.73–3.98)	0.21
T3	0.36	(0.06–1.98)	0.24
T4	1.31	(0.17–9.73)	0.79
N Stage			
N1	1.34	(0.64–2.81)	0.44
N2	0.5	(0.07–3.69)	0.5
Invasive tumor size	1.15	(0.93–1.43)	0.2
Number of positive nodes	1.11	(1.04–1.18)	0.0008
Grade II	1.07	(0.73–1.59)	0.72
Grade III	1.38	(0.82–2.32)	0.23
Stage II	0.84	(0.35–1.93)	0.66
Stage III	3.64	(0.48–27.45)	0.21

*CI* confidence interval, *HR* hazard ratio, *OS* overall survival, *TNBC* triple-negative breast cancer

*HR^+^/HER2^−^ subtype used as reference for comparison with other three subtypes. *P* < 0.05 was considered statistically significant

**Table 3:** Multivariable Cox’s regression analyses for OS in 685 patients with treatments

Variable	Adjusted HR	95% CI	*P* value*
HR^+^/HER2^+^	0.96	(0.63–1.47)	0.84
TNBC	1.5	(1.11–2.04)	0.0091
HER2^+^	2.18	(1.46–3.28)	0.0001
Age at diagnosis > 50	1.94	(1.49–2.53)	< 0.0001
Histology lobular	0.77	(0.51–1.18)	0.24
T Stage			
T2	1.25	(0.85–1.86)	0.26
T3	1.7	(0.71–4.10)	0.24
T4	1.86	(0.83–4.12)	0.13
N Stage			
N1	1.29	(0.88–1.90)	0.2
N2	1.86	(0.94–3.69)	0.07
N3	14.78	(1.86–117.2)	0.01
Invasive tumor size	1.1	(0.996–1.22)	0.059
Number of positive nodes	1.02	(1.006–1.026)	0.0016
Grade II	1.27	(0.93–1.73)	0.14
Grade III	1.4	(0.99–1.98)	0.057
Stage II	1.02	(0.61–1.72)	0.94
Stage III	0.92	(0.38–2.21)	0.85

*HR^+^/HER2^−^ subtype used as reference for comparison with other 3 subtypes. *P* < 0.05 was considered statistically significant

*CI* confidence interval, *HR* hazard ratio, *OS* overall survival, *TNBC* triple-negative breast cancer

In untreated patients, OS rates were similar among HR^+^/HER2^−^, HR^+^/HER2^+^, TNBC, and HER2^+^ subtypes by Kaplan–Meier estimate of the probability (log-rank *P* = 0.983; Fig. 1a). The survival curves overlapped during the entire follow-up time of up to 23.5 years. By contrast, there was a significant association between OS and the subtypes in patients with treatments (log-rank *P* < 0.0001; Fig. 1b). As expected, women with HR^+^/HER2^−^ subtype had best survival, followed by those with HR^+^/HER2^+^, TNBC and HER2^+^ subtypes. Noticeably, survival decreased precipitately within the first 5 years for TNBC and HER2^+^ subtypes, with the decline slowing down during the subsequent 5 years. The difference in OS among subtypes diminished after 10 years and, thereafter, converged, suggesting that the effect of systemic and/or radiation therapy gradually weakened or disappeared.

### Survival by subtype with and without treatments by multivariable analyses

We next evaluated OS for patients in the no-treatment group using multivariable Cox proportional hazards regression model. It was comprised of prognostic factors including not only age at diagnosis, tumor characteristics, number of positive lymph nodes and stage but also the molecular subtypes as covariates (Table 2). As compared with HR^+^/HER2^−^ subtype, adjusted hazard ratio (HR) was 1.1 for HR^+^/HER2^+^ subtype (95% CI 0.55–2.07; *P* = 0.781), 0.62 for HER2^+^ (95% CI 0.29–1.58; *P* = 0.273) and 0.67 for TNBC (95% CI 0.40–1.18; *P* = 0.158) subtypes. Noticeably, survival outcomes of patients with HER2^+^ and TNBC subtypes were not inferior to the HR^+^/HER2^−^ subtype. The risk of breast cancer mortality was 2.2-fold greater for women with age more than 50 years (95% CI 1.35–3.64; *P* = 0.0017), and 1.1 for the number of positive nodes (95% CI 1.04–1.18; *P* = 0.00074). Thus, age and number of positive nodes, rather than breast cancer subtypes, were independent prognostic indicators for unfavorable outcomes in patients who did not receive radiation and systemic therapy.

In women with treatments, HER2^+^ (adjusted HR 2.18; 95% CI 1.46 to 3.28; *P* = 0.0001) and TNBC (1.5; 95% CI 1.11 to 2.04; *P* = 0.0091) subtypes were significantly associated with the decreased survival by multivariable analysis adjusting for age, tumor size and grade, number of positive nodes, stage and breast cancer subtypes (Table 3). Furthermore, N3 status was also associated with a poor prognosis (14.78; 95% CI 1.86 to 117.2). Age and number of positive nodes remain independent factors for unfavorable outcome as were in the untreated group.

## Discussion

Molecular subtypes of breast cancer are extensively investigated surrounding the biological heterogeneity of breast tumors and prognostic relevance in the context of conventional care of management [27]. However, its prognostic value has not been assessed in patients without any type of treatments including systemic and radiation therapy as an independent group. The subtype datasets of “the no systemic therapy” used in several previous studies had included breast tumor samples from patients with radiotherapy, indicative of heterogeneous treatment status [28–30]. For example, a-76 gene signature in node-negative breast cancer was derived from the samples from which 87% of patients received radiotherapy. Mounting evidence indicates that radiotherapy may have systemic effect in addition to its locoregional management [31]. In the participants represented by the CBCTR network, about one-third of patients did not receive systemic treatments nor radiation therapy. Such dataset allows us to assess the biomarkers for bona fide prognosis. The estimate by both Kaplan–Meier and multivariable analyses adjusting for patient and clinicopathologic factors did not detect any significant survival difference among subtypes in untreated patients. van de Vijver et al. had discussed a 70-gene prognosis signature could not predict metastasis-free survival and OS of untreated node-positive patients [20]. Of 97 sporadic breast cancer patient samples that were used to develop the 70-gene prognosis signature, 62 patients received radiation therapy, three received chemotherapy and two with hormonal therapy, which indicated a heterogeneous treatment status [21]. Together, these data are important regarding their potential broad implication in understanding the interplay between treatment outcome and biology of breast cancer subtypes and may have implications in other cancer types [32].

Our results show that patients with HR^+^ subtypes had better outcome with conventional therapy, corroborating with large amount of data in the literature [13, 15, 27, 33]. In the meta-analyses conducted by the Early Breast Cancer Trialists’ Collaborative Group (Oxford Overview), patients with ER^+^ disease significantly benefited from adjuvant endocrine therapy with tamoxifen as well as polychemotherapy with either CMF (cyclophosphamide, methotrexate, and 5-fluorouracil) regimen or anthracycline/taxane-based regimens [3, 34]. In addition, by the Oxford Overview, a greater benefit from radiotherapy was observed in the ER^+^ group [35, 36]. A recent systemic review and meta-analysis in a total of 3798 patients demonstrated that the rate of local–regional control is higher in patients with luminal A subtype than in HER2+ or TNBC patients who received breast conservation therapy followed by radiotherapy [37]. Therefore, HR^+^ or HR^+^/HER2^−^ tumors are not only sensitive to endocrine therapy but also to radiotherapy and chemotherapy. It is the established therapies lasting about 5 years that had led to the better clinical outcome in patients with HR^+^ and/or HR^+^/HER2^−^ disease. Despite these, the risk of breast cancer mortality persists throughout 24 years after primary treatment of ER^+^ breast cancer by a large dataset analysis of clinical trial patients, consistent with our results [38]. Of note, there is no data to directly compare the endocrine therapy alone and chemotherapy alone in HR+ early stage breast cancer since endocrine therapy is given upfront due to its low toxicity and effectiveness. In the context of combination of endocrine treatment and chemotherapy, little (additional) benefit was observed from endochemotherapy compared with endocrine therapy alone in women with low and intermediate Oncotype recurrence scores [39, 40].

We found substantial drops in the survival rates at 5 years in treated relative to untreated women with TNBC and HER2^+^ subtypes (Fig. 1a, b). The worse outcomes within 5 years in basal-like and HER2^+^ subtypes were initially reported in a subgroup of 49 patients with locally advanced tumors and no distant metastases who were treated with neoadjuvant chemotherapy and adjuvant tamoxifen [15]. The subtype phenotype was subsequently confirmed, with variation in significance, by many other studies either with endocrine therapy, chemotherapy or radiotherapy alone, and/or by endochemotherapy, chemoradiotherapy or endo-chemoradiotherapy [27, 28, 38, 41, 42]. A historic cohort study also showed the outcome difference that was mostly observed within the first 5 years of follow-up among breast cancer subtypes [19]. Further, HER2^+^ and TNBC subtypes were less effectively responded to radiation therapy relative to the luminal subtypes by a recent systemic review and meta-analysis in four clinical breast cancer subtypes, and this phenomenon was observed in other studies as well [37, 43]. Moreover, despite initial sensitivity to neoadjuvant chemotherapy, patients with basal-like and HER2^+^ tumors paradoxically had early relapse, and worse overall and distant disease-free survivals [44]. Altogether, these data may have prompted a need for re-examination of treatment approaches for certain breast cancer subtypes such as TNBC. On the other hand, HER2-targeted therapy likely has shifted the outcome of patients with HER2^+^ subtype, which the population-based estimate is ongoing [45].

It has been shown that triple-negative and HER2^+^ primary breast tumors exhibited higher frequency of expression of γH2AX, a component in the ATM/H2AX DNA damage response complex that facilitates the DNA damage repair [23]. The data that constitutive expression of γH2AX at diagnosis was associated with worse survival in patients who received chemotherapy provided a molecular mechanism of action of resistance [46]. In addition, a large decrease versus minor reduction of γH2AX was associated with better response to neoadjuvant chemotherapy in TNBC [44].

It is worth to note that the size of this investigation population was relatively small, compared to those of a similar type. Despite the limitation, our research represents a first step, with significance, to address the question of the molecular subtypes of breast cancer for prognosis in patients with and without conventional treatments as distinct entities. Validation of the association between the breast cancer subtypes and treatment outcomes is warranted in other retrospective studies and clinical trials.

## Conclusions

The results through 23.5 years of clinical follow-up provided an evidence of the impact of conventional therapy to the differential survival outcomes in treated versus untreated patients with distinct breast cancer subtypes. In addition, patients in this study represented the general breast cancer population who received their care at community hospitals. Our results, if validated by other studies and clinical trials, may have an implication in prompting the re-examination of treatment modalities such as chemotherapy that currently apply to all TNBC and HER2^+^ patients.

## Electronic supplementary material

Below is the link to the electronic supplementary material.

Supplementary material 1. Additional file 1: Table S1 Age and clinicopathologic variables at diagnosis among breast cancer subtype by treatment. (DOCX 20 KB)

## Abbreviations

CI: Confidence interval
ER: Estrogen receptor
HR: Hormone receptor; hazard ratio
HER2: Human epidermal growth factor receptor-2
PR: Progesterone receptor
TNBC: Triple-negative breast cancer
OS: Overall survival
