# Supplementary material for: Systemic treatment and radiotherapy, breast cancer subtypes, and survival after long-term clinical follow-up
Source: Breast Cancer Res Treat. 2019 Feb 12;175(2):287–95. doi: 10.1007/s10549-019-05142-x (PMC6533413; doi:10.1007/s10549-019-05142-x)
Supplement: Supplementary file 1 — Supplementary material 1. Additional file 1: Table S1 Age and clinicopathologic variables at diagnosis among breast cancer subtype by treatment. (DOCX 20 KB) [file 10549_2019_5142_MOESM1_ESM.docx]

**Additional file 1:**

**Table S1** Age and clinicopathologic variables at diagnosis among breast cancer subtype by treatment

| Variable/Subtype | Total (685)  No. (%) | HR^+^/HER2^−^ (468)  No. (%) | HR^+^/HER2^+^ (58)  No. (%) | TNBC (109)  No. (%) | HER2^+^ (50)  No. (%) | *P* value* |
| --- | --- | --- | --- | --- | --- | --- |
| Age at Diagnosis  ≤50  >50  Histology  Ductal  Lobular  T Stage  T1  T2  T3  T4  N Stage  N0  N1  N2  N3  Nx  Tumor size  ≤2  2 – 5  >5  Missing  N of positive nodes  0  1 – 3  4 – 9  ≥10  Missing  Tumor grade  I  II  III  Stage  I  II  III  ER status  Negative  Positive  PR status  Negative  Positive  HER2 status  Negative  Positive | 220 (32.1%)  465 (67.9%)  623 (90.9%)  62 (9.05%)  346 (50.5%)  213 (31.1%)  79 (11.5%)  47 (6.86%)  320 (46.7%)  314 (45.8%)  49 (7.15%)  1 (0.146%)  1 (0.146%)  347 (50.7%)  243 (35.5%)  94 (13.7%)  1 (0.1%)  320 (46.8%)  168 (24.6%)  120 (17.5%)  76 (11.1%)  1 (0.001%)  141 (20.6%)  326 (47.6%)  218 (31.8%)  237 (34.6%)  295 (43.1%)  153 (22.3%)  191 (27.9%)  494 (72.1%)  237 (34.6%)  448 (65.4%)  577 (84.2%)  108 (15.8%) | 130 (27.8%)  338 (72.2%)  408 (87.2%)  60 (12.8%)  256 (54.7%)  135 (28.8%)  49 (10.5%)  28 (5.98%)  231 (49.4%)  204 (43.6%)  31 (6.62%)  1 (0.214%)  1 (0.214%)  257 (55%)  152 (32.5%)  58 (12.4%)  1 (0.1%)  231 (49.5%)  114 (24.4%)  75 (16.1%)  47 (10.1%)  1 (0.001%)  133 (28.4%)  248 (53%)  87 (18.6%)  175 (37.4%)  197 (42.1%)  96 (20.5%)  26 (5.56%)  442 (94.4%)  67 (14.3%)  401 (85.7%)  468 (100%)  0 (0%) | 25 (43.1%)  33 (56.9%)  57 (98.3%)  1 (1.72%)  28 (48.3%)  16 (27.6%)  9 (15.5%)  5 (8.62%)  29 (50%)  26 (44.8%)  3 (5.17%)  0 (0%)  0 (0%)  28 (48.3%)  19 (32.8%)  11 (19%)  0 (0%)  29 (50%)  17 (29.3%)  3 (5.17%)  9 (15.5%)  0 (0%)  3 (5.17%)  31 (53.4%)  24 (41.4%)    23 (39.7%)  21 (36.2%)  14 (24.1%)  6 (10.3%)  52 (89.7%)  11 (19%)  47 (81%)  0 (0%)  58 (100%) | 48 (44%)  61 (56%)  108 (99.1%)  1 (0.917%)  47 (43.1%)  36 (33%)  16 (14.7%)  10 (9.17%)  43 (39.4%)  57 (52.3%)  9 (8.26%)  0 (0%)  0 (0%)  47 (43.1%)  43 (39.4%)  19 (17.4%)  0 (0%)  43 (39.4%)  27 (24.8%)  26 (23.9%)  13 (11.9%)  0 (0%)  4 (3.67%)  34 (31.2%)  71 (65.1%)  29 (26.6%)  49 (45%)  31 (28.4%)  109 (100%)  0 (0%)  109 (100%)  0 (0%)  109 (100%)  0 (0%) | 17 (34%)  33 (66%)  50 (100%)  0 (0%)  15 (30%)  26 (52%)  5 (10%)  4 (8%)  17 (34%)  27 (54%)  6 (12%)  0 (0%)  0 (0%)  15 (30%)  29 (58%)  6 (12%)  0 (0%)  17 (34%)  10 (20%)  16 (32%)  7 (14%)  0 (0%)  1 (2%)  13 (26%)  36 (72%)  10 (20%)  28 (56%)  12 (24%)  50 (100%)  0 (0%)  50 (100%)  0 (0%)  0 (0%)  50 (100%) | 0.002  <0.001  0.024  0.677  0.004  0.014  <0.001  0.062  <0.001  <0.001  <0.001 |

**P* < 0.05 was considered statistically significant.

CI, confidence interval; ER, estrogen receptor; HER2, human epidermal growth factor receptor 2; HR, hazard ratio; PR, progesterone receptor; No., number; OS, overall survival; TNBC, triple-negative breast cancer.
